# Measurement and Clinical Significance of Biomarkers of Oxidative Stress in Humans

**DOI:** 10.1155/2017/6501046

**Published:** 2017-06-18

**Authors:** Ilaria Marrocco, Fabio Altieri, Ilaria Peluso

**Affiliations:** ^1^Department of Biochemical Sciences “A. Rossi Fanelli”, Sapienza University, P.le A. Moro 5, 00185 Rome, Italy; ^2^Center for Food and Nutrition, Council for Agricultural Research and Economics (CREA-AN), Via Ardeatina 546, 00178 Rome, Italy

## Abstract

Oxidative stress is the result of the imbalance between reactive oxygen species (ROS) formation and enzymatic and nonenzymatic antioxidants. Biomarkers of oxidative stress are relevant in the evaluation of the disease status and of the health-enhancing effects of antioxidants. We aim to discuss the major methodological bias of methods used for the evaluation of oxidative stress in humans. There is a lack of consensus concerning the validation, standardization, and reproducibility of methods for the measurement of the following: (1) ROS in leukocytes and platelets by flow cytometry, (2) markers based on ROS-induced modifications of lipids, DNA, and proteins, (3) enzymatic players of redox status, and (4) total antioxidant capacity of human body fluids. It has been suggested that the bias of each method could be overcome by using indexes of oxidative stress that include more than one marker. However, the choice of the markers considered in the global index should be dictated by the aim of the study and its design, as well as by the clinical relevance in the selected subjects. In conclusion, the clinical significance of biomarkers of oxidative stress in humans must come from a critical analysis of the markers that should give an overall index of redox status in particular conditions.

## 1. Introduction

The redox equilibrium is important in preserving the correct functionality of cellular vital functions [1]. Oxidative stress is defined as the imbalance in the redox characteristics of some cellular environment which can be the result of either biochemical processes leading to the production of reactive species, exposure to damaging agents (i.e., environmental pollutants and radiations), or limited capabilities of endogenous antioxidant systems [2–4]. Reactive oxygen and nitrogen species (ROS/RNS) produced under oxidative stress are known to damage all cellular biomolecules (lipids, sugars, proteins, and polynucleotides) [5, 6]. Thus, several defense systems have been involved within the cells to prevent uncontrolled ROS increase. These systems include nonenzymatic molecules (glutathione, vitamins A, C, and E, and several antioxidants present in foods) as well as enzymatic scavengers of ROS, with superoxide dismutase (SOD), catalase (CAT), and glutathione peroxidase (GPX) being the best-known defense systems [1].

Mitochondria are the predominant source of ROS in all cell types [7]. Superoxide (O_2_^•−^) is mainly generated at the level of the mitochondrial electron transport chain and can be converted to hydrogen peroxide (H_2_O_2_) by SOD or undergo spontaneous dismutation [1]. In the presence of transition metal ions, for example, iron and copper ions, H_2_O_2_ can generate via Fenton reaction the highly reactive hydroxyl radical (HO^•^). Reactive species may also be enzymatically produced by xanthine oxidase (XO), uncoupled nitric oxide synthases (NOS), and NADPH oxidase (NOX). ROS production is related not only to cell damage or death, but physiological and signalling roles for ROS have also been ascertained. Reactive species are the principal source of defensive pro-oxidants generated in the respiratory burst of neutrophils [8, 9]. Upon activation, neutrophils produce various ROS via myeloperoxidase (MPO) and RNS via inducible nitric oxide synthase (iNOS). MPO catalyzes the H_2_O_2_-dependent formation of hypochlorous acid (HClO) while iNOS produces nitric oxide (NO^•^), which then reacts with O_2_^•−^ to form peroxynitrite (ONOO^−^) [10]. NOX associated with cell membrane catalyzes the generation of superoxide radicals that play a physiological role in cancer invasion, hypoxia, and integrin signaling [11–13]. Furthermore, ROS can modulate the expression of several genes through the redox regulation of the nuclear factor-erythroid 2-related factor 2 (Nfr2) and the nuclear factor kappa-light-chain-enhancer of activated B cells (NF-kB) [1, 14]. A concerted modulation of these pathways has been suggested in inflammation and carcinogenesis [14].

During the past decade, research has revealed a widespread involvement of oxidative stress in a number of disease processes, including cancer, cardiovascular disease (CVD), atherosclerosis, diabetes, arthritis, neurodegenerative disorders, and pulmonary, renal, and hepatic diseases [1, 5, 15–23]. These pathologic states have increased incidence with age, and oxidative stress is believed to be a major factor in ageing and ageing-associated diseases [24–26]. Thus, oxidative stress markers are important tools to assess the biological redox status, disease state and progression, and the health-enhancing effects of antioxidants in humans. Identifying markers of oxidative stress has been the focus of many studies, and several markers from various biomolecule sources have been proposed over the past decades. However, for some of them, there is a lack of consensus concerning validation, standardization, and reproducibility. We aim to discuss the major bias of these methods.

## 2. Measurement of Reactive Species in Leukocytes and Platelets by Flow Cytometry

In humans, under physiological conditions, ROS and RNS generated by leukocytes, through NOX and iNOS, have a role in the innate immune response to infection [8, 9]. However, ROS and RNS can induce lipid peroxidation of polyunsaturated fatty acids (PUFAs), which propagate via peroxyl radicals (ROO^•^) within the membrane, as well as in the low-density lipoproteins (LDL) [5, 2721]. In particular, in the context of metabolic syndrome and chronic inflammation, the oxidized LDL (oxLDL) activate leukocytes and/or platelets to produce ROS and RNS [27–29].

The direct quantification of ROS/RNS is a valuable and promising biomarker that can reflect the disease process. However, given the short half-life of these species, their measurement in biological systems is a complex task. Approaches include electron spin resonance, fluorescence magnetic resonance, and mass spectrometry techniques [30, 31], but their use has been limited to cell cultures and other in vitro applications. Although free radicals' production can be measured by spectrophotometric or luminescence methods [32, 33], all extracellular free radicals' measurements are deeply affected by cell count and viability.

On the other hand, flow cytometry is one of the most powerful tools for single-cell analysis of the immune system [34] and it is routinely used in the diagnosis and progression evaluation of blood cancers [35–38] and human immunodeficiency virus (HIV) infection [39–41]. In addition to the role of oxidative burst evaluation by flow cytometry in the diagnosis of chronic granulomatous disease [42], this instrumentation has been used for many years to evaluate oxidative burst in many conditions, such as autoimmune neutropenia [43] and asymptomatic HIV+ individuals [44].

Many fluorescent probes for the detection of reactive species have been developed in the last years, with a different degree of specificity and sensitivity [45]. The fluorescent probes used for the detection of reactive species in blood cells via flow cytometry are summarized in Table 1.

For instance, intracellularly converted diacetate derivatives of probes such as dihydrochlorofluorescein diacetate (DCFH-DA), 4,5-diaminofluorescein diacetate (DAF-2 DA), and 4-amino-5-methylamino-2′,7′-difluorofluorescein diacetate (DAF-FM DA) have widely been used for ROS/RNS detection [32, 33, 45–47]. Once taken up by cells, these probes are hydrolyzed by intracellular esterases, generating the nonfluorescent and membrane-impermeable DCFH, DAF-2, or DAF-FM. Subsequent oxidation by ROS/RNS results in the formation of the fluorescent 2′,7′-dichlorofluorescein (DCF) and triazolofluoresceins (DAF-Ts), respectively.

DCFH, the more commonly used probe, does not directly react with H_2_O_2_ to form the fluorescent product. DCFH can be instead oxidized to DCF by several one-electron-oxidizing species including HO^•^ radicals, products formed from peroxidase or heme proteins reacting with H_2_O_2_, HClO, and nitrogen dioxide (NO_2_^•^) generated by myeloperoxidase and peroxynitrite decomposition. DCFH oxidation can also be promoted by Fe^2+^ in the presence of O_2_ or H_2_O_2_. The 1-electron oxidation of DCFH generates the DCF semiquinone anion radical (DCF^•−^). This intermediate can rapidly react with O_2_ to form O_2_^•−^, which in turn can dismutate yielding additional H_2_O_2_ and establishing a redox-cycling mechanism that leads to an artificial amplification of the fluorescence signal [46].

While DCFH is used in both platelets and leukocytes, dihydrorhodamine 123 (DHR123) and hydroethidine (HE) are used only in the evaluation of the oxidative burst by polymorphonuclear leukocytes (PMN) (Table 1).

DHR123 is an uncharged nonfluorescent probe that passively diffuses across cell membranes and is converted upon oxidation to the fluorescent membrane-impermeant rhodamine 123 (Rho123), which predominantly localizes in the mitochondria [32, 33, 45, 47]. HE passively diffuses into cells and is preferentially oxidized by O_2_^•−^ to ethidium, which results in intercalation in DNA and consequently a significant enhancement of its red fluorescence intensity [32, 33, 45, 47].

The 4,4-difluoro-5-(4-phenyl-1,3-butadienyl)-4-bora-3a,4a-diaza-s-indacene-3-undecanoic acid (C11-BODIPY^581/591^) probe is the only lipophilic probe used to evaluate ROS in leukocytes and platelets [48, 49]. C11-BODIPY^581/591^ is a derivatized 11-carbon fatty acid in which the boron dipyrromethene difluoride (BODIPY) core is substituted by a phenyl group via a conjugated diene [50, 51]. This conjugated diene interconnection is oxidation sensitive, and when oxidized by HO^•^ or ROO^•^, disruption and shortening of the conjugated electron resonance structures between the phenyl group and the BODIPY core shifts C11-BODIPY^581/591^'s fluorescence from red to green [50, 51]. Conversely, ONOO^−^ induces not only oxidation but also nitration of BODIPY, reducing red fluorescence but not necessarily increasing green fluorescence [52]. Although excimers of the oxidized form are red fluorescent, labelling conditions up to 30 *μ*M provides sufficient staining of the plasma and organelle membranes well below the range in which self-quenching or excimer formation occurs [51]. Therefore, excimers do not interfere with the fluorescence of BODIPY and the measured red signal depends only on the reduced form of the probe. Furthermore, neither C11-BODIPY^581/591^ nor its oxidation products are able to spontaneously leak from the lipid bilayer [51] and the ratio of oxidized to nonoxidized C11-BODIPY^581/591^ can be used to normalize probe incorporation in cells of different size (lymphocytes, monocytes, and granulocytes) [49]. Only hemolysis and antioxidants, in particular the end-product of purine metabolism, uric acid (UA), could bias the measurement of ROS generation [49, 53].

On the contrary, when analyzing the results of intracellular probes, many factors must be taken into account (Table 1).

Ethidium displacement by molecules, such as chemotherapeutics [54] or flavonoids [47], could decrease the ethidium fluorescence signal, making the interpretation of data difficult.

Artefactual amplification of the fluorescence intensity has been suggested to occur via intermediate radicals for both DCF and DHR [46], whereas the presence of quenching and reducing antioxidants could either decrease [55] or increase [56] the oxidation of probes without affecting ROS production. Heme proteins and reduced iron (Fe^2+^) have been shown to oxidize DCFH, and the suitability of DCFH-DA for measuring intracellular ROS is increasingly being questioned [46].

In addition to the aforementioned limitations, the fluorescence signal is dependent not only on the oxidation of the probe but also on its concentration. In this context, multidrug resistance- (MDR-) mediated transport has low substrate specificity. Multidrug resistance-related protein 2- (MRP2-) mediated DCF extrusion has been reported in human leukocytes [57], and it is well known that Rho123 can be extruded by the MDR [47]. The inclusion of H2DCF-DA in the dilution buffer in order to avoid dye leakage has been suggested [58]. However, overloading with probe generates cell morphology changes and artifacts in platelets [59, 60]. In this context, it should be pointed out that lyophilic derivatives of intracellular fluorescent probes are substrates of P-glycoprotein (Pgp) and MRP1 [47]. Furthermore, MDR expression is affected by intracellular variation of glutathione (GSH) [61] and oxidative stress [62–65], as well as by various dietary factors [66–69], inflammatory cytokines [70–72], disease states [73–75], and drugs [76–81]. In particular, aspirin, indomethacin, and ibuprofen are substrates for MRP4 [76] and may interfere with fluorescent probe staining. Most importantly, aspirin treatment over a period of 15 days significantly increased MRP4 mRNA and protein expression in platelets of healthy volunteers [78]. MRP4 is involved in the storage of cyclic nucleotides in dense granules [82–84], and MRP4 inhibition impairs platelet aggregation [85]. Besides the aforementioned effects, MRP4 also has a role, together with MRP1 [86], in the efflux of leukotrienes [87]. Therefore, in addition to the potential confounding effect on the fluorescence signal [88], the presence of intracellular probes per se could reduce platelet activation in vitro.

In addition, intracellular esterase activity was shown to be impaired in damaged platelets and highly correlated with ADP-induced aggregation [89], whereas plasma esterases [59, 60] and/or inhibition of esterases [47] could potentially interfere with probe staining and fluorescence signal intensity when using DCFH-DA, DAF-2 DA, and DAF-FM DA in whole blood and platelet-rich plasma (PRP) methods.

Despite the fact that whole blood methods provide more physiologically relevant data when evaluating ROS production in leukocytes [32], washing impairs ADP-induced aggregability of platelets [90] and alters their structure [91], whereas ethylenediaminetetraacetic acid (EDTA) and citrate increase DCFH oxidation [56].

Moreover, whole blood, PRP, and platelet-poor plasma [92] also contain XO, and therefore, UA may be produced during the incubation period with ROS-inducers, potentially falsifying results. Urate crystals induced oxidative burst [27] and the activation and lysis of platelets in vitro [93, 94].

With this in mind, it is well known that there is an increased platelet destruction and production in some patients with primary gout [95, 96] and that platelet apoptosis and microparticles derived from platelets, erythrocytes, leukocytes, and/or endothelial cells are higher in subjects with CVD [97–99], dyslipidemia [100], and metabolic syndrome [101]. On the other hand, lipid-lowering treatment [100] and the XO inhibitor febuxostat [102] were shown to decrease microparticle count. Gender differences have been reported for microparticle count. Specifically, higher levels of microparticles have been found in women compared with men [103]. Endotoxin induced the formation of platelet microparticles [104], introducing potential confounding factors in conditions of increased levels of lipopolysaccharide, such as the postprandial state [105] and metabolic [106] and inflammatory diseases [107]. Spontaneous activation, generating both microparticles and inducing microaggregation of platelets, occurs in type 2 diabetic patients [108], increases with age in healthy subjects [109], and is affected by blood collection and processing procedures [109, 110]. On the other hand, platelet aggregates with leukocytes are a marker of activated platelets in CVD patients [111–114], potentially reducing the platelet count in PRP. All these factors must be taken into account when evaluating data from case-control studies that compared ROS-production in unstimulated samples of disease and healthy subjects.

**Figure 1:**
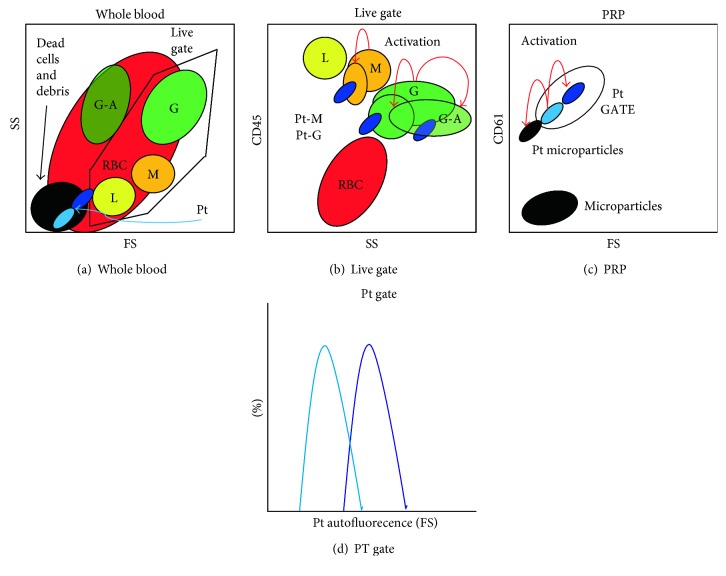
Gating strategies in the measure of free-radical production by flow cytometry. Different leukocytes populations (lymphocytes: L, monocytes: M, and granulocytes: G) in whole blood can be identified by CD45 (b) in the live gate assigned in the forward scatter (FS) and side scatter (SS) dot plot (a) by excluding dead cells and debris. Red blood cells (RBC) can be excluded as CD45 negative (b). Platelets (Pt) can be identified by CD61 in platelet-rich plasma (PRP) (c). In activated samples, platelet microparticles (c) and leukocyte-platelet aggregates (b: Pt-G and Pt-M) are formed and Pt-G are more prone to apoptosis (G-A). After platelet activation, FS increases due to platelet aggregation inducing an increase in autofluorescence (d).

The combination of fluorescently labeled antibodies against targets such as the pan-leukocyte marker CD45 [49] and the platelet marker CD61 [48] and/or physical properties such as size (FS: forward scatter) and internal complexity (SS: side scatter) can identify different leukocyte populations and platelets (Figure 1).

In activated samples, platelet microparticles [103, 104, 115], platelet aggregates [116], and leukocyte-platelet aggregates [117, 118] are formed (Figure 1). In particular, platelet activation in whole blood induces the formation of platelet conjugates with granulocytes or monocytes [119] and leukocyte aggregates with platelets are more prone to apoptosis after in vitro activation (Figure 1) [117].

Regarding the normalization strategies, stimulation indexes calculated from the mean intensity fluorescence (MIF) values and expressed as fold change relative to unstimulated samples have been suggested for evaluating the production of ROS in both granulocytes [120, 121] and platelets [122, 123]. However, these methods do not take into account probe leakage nor autofluorescence differences. While it is well known that autofluorescence generates false-positive monocytes [124], this aspect is neglected in platelet assays. Despite controversy regarding the relationship between CVD and platelet size, measured as mean platelet volume (MPV) or FS [97, 125, 126], it is well known that FS increases after platelet activation [127] and that large and small platelet subpopulations have different autofluorescence profiles [128] (Figure 1). Consequently, differences in autofluorescence in unstimulated and stimulated samples imply that stimulation indexes do not necessarily measure ROS production. Therefore, it must always be taken into account that the fluorescence signals and not the radicals are measured and that the oxidation of the probe is not always related to ROS production. Overall, the reviewed potential bias and confounding factors suggest that accurate gating and normalization strategies must be applied in order to avoid misinterpretation of the results.

## 3. Markers Based on ROS-Induced Modifications

In addition to the measure of free-radical production, a different approach is measuring stable markers that may reflect a systemic or tissue-specific oxidative stress. Such molecules are modified by the interaction with ROS in the microenvironment [129] (Table 2).

Lipids, DNA, and proteins are examples of molecules that can be modified by excessive ROS in vivo (Table 2) [129]. Some of these modifications are known to have a direct effect on the function of target molecules, such as the inhibition of an enzymatic function, but other modifications just reflect the local degree of oxidative stress. This influences the clinical applicability of several oxidative stress markers since the functional significance or the causal role of oxidative modifications on biological functions is a key characteristic for the validity of a biomarker (Table 2).

While measures of oxidative stress in spinal cord [130] and tissues [131, 132] are restricted to particular disease conditions, venous blood and urinary samples are the most commonly used in clinical practice. In addition to urinary samples [133–135], other noninvasive and low-cost tools for the screening of oxidative stress, such as salivary [136–138] or exhaled breath [139–141] analysis, have been proposed. However, it has been reported that creatinine urinary markers are not suitable in patients with impaired renal function [135]. Therefore, the validity of a biomarker depends on the choice of the sample that should be dictated by subjects' characteristics and the best cost-benefit ratio.

### 3.1. Lipid Oxidation Products

Lipid oxidation end product determination is a widely used marker of oxidative stress.

The presence of unsaturated double bonds makes PUFAs, mainly arachidonic acid (AA), highly susceptible to oxidative damage in the presence of ROS or free radicals [5]. Lipids peroxidation may also occur through enzymatic reactions, catalyzed by lipooxygenase and cyclooxygenase (COX), which oxidize AA into prostaglandins, prostacyclin, tromboxane, and leukotrienes. Free radical-mediated oxidation involves an autocatalytic chain reaction triggered by ROS, mainly HO^•^ and ROO^•^, which catalyze a hydrogen-atom subtraction at the unsaturated bonds generating a carbon radical that can further react with oxygen producing a lipid peroxyl radical. The chain reaction proceeds with lipid peroxyl radical acting as chain-carrying radicals and the formation of lipid hydroperoxydes. In the presence of transition metals, lipid hydroperoxides may generate lipid alkoxyl and ROO^•^ as well as HO^•^, which can further sustain the chain oxidation reaction to produce short-chain oxidation products, including a variety of different aldehydes, alkanes, and alkenes. Malondialdehyde (MDA) and 4-hydroxy-2-nonenal (HNE) represent the most investigated end product of lipid oxidation [142]. HNE can be detected by high-performance liquid chromatography (HPLC) directly or as a derivatized product with 2,4-dinitrophenylhydrazine or 1,3-cyclohexanedione, by gas chromatography coupled with mass spectroscopy (GC-MS), and by means of immunological techniques using specific anti-HNE antibodies [142–144]. However, when 4-HNE aldehydes were determined using GC-MS system, they were significantly different in plasma and urine of patients with rheumatoid arthritis compared to healthy subjects, but differences between patients with low and high disease activity can be detected only in plasma samples, suggesting that only this sample is useful to monitor the progression of this autoimmune disease [145].

MDA, alkenals, and alkadienals constitute the thiobarbituric acid reactive substances (TBARS), which can react with two equivalents of thiobarbituric acid (TBA) to give a pink adduct complex, easily measured by a colorimetric or fluorimetric assay (Table 2). Despite TBA test for MDA determination being the most frequently used method to evaluate lipid peroxidation, it shows several pitfalls and has been criticized as being too unspecific and prone to artifacts [146–148]. TBA can react with several compounds, including sugars, amino acids, bilirubin, and albumin, producing interferences in the measurement (Table 2). There is a further MDA generation, which occurs during the procedure itself that may be prevented by adding an antioxidant, like butyl hydroxytoluene (BHT), and by reducing the heating time. An additional pitfall is the interference of hemolysis that falsely increases the measured MDA levels (Table 2). Thus, many protocols and modifications of the TBA test are available in the literature, and while direct MDA-TBA adduct measurement has a low significance, the determination by HPLC combined with UV or fluorescence detection is a more reliable and reproducible method [149–151]. Despite the methodological bias, MDA measurement could have clinical relevance due to the potential pathogenic role of MDA on to the induction of IL-17 producing cells [152] and a possible link between lipid-peroxidation and T-helper 17 (Th17) cell-mediated diseases, such as inflammatory bowel diseases [153].

F2-isoprostanes (F2-IsoPs), chemically stable prostaglandin-like isomers generated by the reaction of polyunsaturated fatty acids in membrane phospholipids and free radicals or ROS, represent another reliable marker assessing oxidative stress status in vivo [154–156]. In fact, they are initially formed in lipid membranes as a consequence of oxidative stress and then released in free form by phospholipase action. F2-IsoPs are unaffected by lipid content in diet and thus their measurement in biological fluids as well as exhaled breath condensate can provide an estimation of total body production, whereas measurement of F2-IsoPs esterified in tissues of interest can provide information to localize and quantify the specific oxidative stress. Despite these observations, the utility of F2-IsoPs as biomarkers of oxidative stress is highly limited since their reliable quantification is costly requiring gas/liquid chromatography coupled with mass spectroscopy techniques (HPLC/GC-MS). It must be taken into account that also measures of both MDA and 15(S)-8-iso-PGF(2alpha) by GC-MS/MS in plasma samples may be markedly compromised by hemolysis [154]. Immunoassay techniques, based on specific antibodies, are under development, but their application is limited since the obtained results do not correlate well with mass spectrometry determination [155–157]. In addition to the methodological considerations, it must be taken into account that in some inflammatory conditions, the enzymatic product of arachidonic acid prostaglandin F2*α* (PGF2*α*) must be evaluated with nonenzymatic oxidation products (F2-IsoPs) in different tissues [158]. In fact, it has been recently reported that PGF2*α* levels, but not F2-IsoPs, were higher in cerebrospinal fluid of patients with multiple sclerosis (compared with controls); however, in plasma, both F2-IsoPs and PGF2*α* were lower in patients with progressive disease and decreased with increasing disability score [158]. A good approach could be to study the profiling of eicosanoid metabolome, as recently suggested in animal models of rheumatoid arthritis [159, 160].

### 3.2. Markers of DNA Oxidation

Oxidation of DNA components by ROS/RNS is the major source of induced DNA damages leading to several types of DNA modifications including nucleotide oxidation, strand breakage, loss of bases, and adduct formation [161, 162]. The HO^•^ radical can react with all purine and pyrimidine bases, as well as deoxyribose backbone, generating various products, the most common one being 7,8-dihydroxy-8-oxo-2′-deoxyguanosine (8oxodG) [163].

Oxidatively generated lesions can lead to decomposition in base fragments and the formation of carbon-centered radicals, which give rise, in most cases, to DNA strand breaks. Exposure of DNA to RNS can promote deamination of DNA bases and conversion of guanine into xanthine, oxanine, and 8-nitroguanine, which is rapidly lost from DNA by spontaneous depurination. The major end products of HClO include 5-chlorocytosine and 5-chlorouracil. These modified bases have been detected at sites of inflammation and are indicative of HClO-mediated DNA damage in vivo [164].

DNA damage may also be caused by the attack of reactive products resulting from ROS-induced modifications of other molecules, such as lipids. In this case, etheno-DNA adducts, such as 1,N(6)-etheno-2′-deoxyadenosine (*ε*dA) and 3,N(4)-etheno-2′-deoxycytidine (*ε*dC), are formed and can be used as biomarkers of oxidative stress [165] and may serve as potential markers for assessing progression of inflammatory cancer-prone diseases [166]. Elevated etheno-DNA adducts were found in tissues of patients suffering from chronic inflammatory processes [167] while increased levels of urinary *ε*dA were observed in subjects and workers exposed to diesel engine exhaust [168]. Etheno-DNA adducts can be measured by HPLC/MS-based techniques [165, 169].

It has been estimated that several thousands of 8-oxodG lesions may form daily in a mammalian cell, representing 5% of all oxidative lesions, and for this reason, 8-oxoG is the most commonly used biomarker of DNA oxidation to measure oxidative stress [170–172]. However, analysis of 8-oxodG and other oxidized purines and pyrimidines has been hampered for a long time by the occurrence of several drawbacks associated with their measurement. Optimized assays are now available, and the most reliable is represented by chromatography coupled with mass spectroscopy, even if commercial ELISA assays based on specific antibodies are available [173, 174]. Although ELISA methods are less specific compared to HPLC with electrochemical detection (HPLC-ECD) and HPLC/GC-MS, some kits with specific antibodies resulted appropriate for urine samples [175].

The oxidized nucleotides are excreted into the urine, and their measurement has been proved to be predictive of the development of several diseases. High level of DNA oxidation, measured as urinary excretion of 8oxodG, is predictive for the risk of breast and lung cancer, atherosclerosis, and diabetes [176–179]. RNA oxidation, measured as 7,8-dihydroxy-8-oxoguanosine (8oxoGuo), has been recently introduced as a marker of diseases, particularly neurodegeneration and diabetes, and high level of RNA oxidation has been also associated with breast cancer development in diabetic females [180].

### 3.3. Protein Oxidation Products

**Figure 2:**
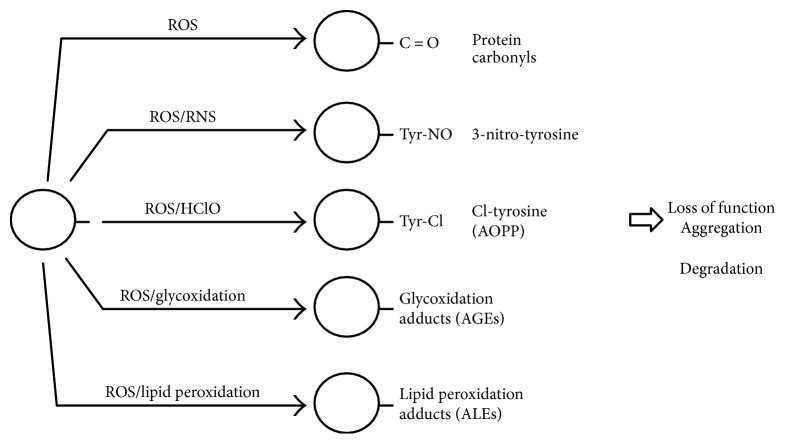
Irreversible oxidative modifications of proteins. AGEs: advanced glycation end products; ALEs: advanced peroxidation end products; AOPP: advanced oxidation protein products; HClO: hypochlorous acid; RNS: reactive nitrogen species; ROS reactive oxygen species.

Proteins represent a wide target for ROS and RNS generated under normal or oxidative stress conditions and can be considered as general scavengers of these species. Several amino acidic residues can undergo oxidative modifications including oxidation of sulphur-containing residues, hydroxylation of aromatic and aliphatic groups, nitration of tyrosine residues, nitrosylation and glutathionylation of cysteine residues, chlorination of aromatic groups and primary amino groups, and conversion of some amino acid residues to carbonyl derivatives [181, 182] (Figure 2).

Oxidation can also lead to the cleavage of the polypeptide chain and to the formation of cross-linked protein aggregates [183, 184] (Figure 2).

Oxidation of iron-sulphur centers by O_2_^•−^ is irreversible and leads to enzyme inactivation. In addition, metals bound to the protein can generate, through the Fenton reaction, HO^•^ radicals that rapidly oxidize the neighbor amino acid residues of the protein [185].

If the oxidative modifications of protein residues are not properly repaired or removed, they could affect the three-dimensional structure and physicochemical properties of the protein that may also become toxic.

Irreversible modifications of proteins include carbonylation, nitrosilation, breaking of the histidine and tryptophan rings, and hydrolysis of the peptide bond in the presence of proline [186]. The latter mainly occurs in the collagen, rich in proline and hydroxyproline, which is particularly damaged under oxidative stress conditions [187].

Determination of protein oxidation has a biological significance and a good clinical relevance. A specific profile of oxidized proteins may be formed as a consequence of different oxidative stress or age-related diseases [188–190]. Biological significance of protein oxidation may also result from its chemical stability and high yield formation. Sample availability is an important factor that limits the reliability of a biomarker. Protein oxidation may be determined in blood and urine samples, although determination in specific tissue or cell samples may give more precise information. It must be noted that protein oxidation may occur during the analytical process thus generating some artefacts [191]. The rates of oxidation reactions are critically dependent on the sample temperature, its physical form, and the presence of oxygen and catalysts (metal ions and light) [192]. For these reasons, measurement of protein oxidation may be a useful marker, as long as it is characterized by a high reproducibility, sensitivity, and specificity.

Several methods have been developed for the detection of the different kinds of protein modifications. However, the biological and clinical relevance of protein oxidation as a biomarker is still limited by the availability of methodologies able to identify and quantify specific protein oxidative modifications.

#### 3.3.1. Protein Carbonyls, ALEs, and AGEs

Carbonyl groups can be generated by many different mechanisms, as the oxidative cleavage of the protein backbone, in particular at the level of glutamyl side chains, and the oxidative deamination of lysine. Also, the attack of HO^•^ radicals on proline, lysine, arginine, and threonine side chains generates carbonyl groups [193].

The measure of carbonyl levels in proteins is the most widely used marker of oxidative protein damage, and tissues injured by oxidative stress generally contain increased concentrations of carbonylated proteins [186, 194]. Moreover, this biomarker has some advantages in comparison with the measurement of other oxidation products because of the relative early formation and the relative stability of carbonylated proteins. Protein carbonyl levels increase with age and are elevated in several pathologic conditions including neurodegenerative diseases, obesity, or diabetes [195, 196].

Methods based on ELISA and HPLC are the most used in clinical assessments because of high throughput and standardization. Detection of protein carbonyl groups generally involves the derivatization of the CO group with 2,4-dinitrophenylhydrazine (DNPH) with the formation of a stable dinitrophenyl (DNP) hydrazone product. The latter can be detected by several methods which include the direct spectrophotometric measurement of DNP adducts, as well as more specific techniques based on anti-DNP antibodies, like ELISA, Western blot after one-dimensional or two-dimensional electrophoretic separation, immunohistochemistry, and HPLC [197–199].

Functional groups of proteins can react with several products resulting from the ROS-induced oxidation of PUFAs and carbohydrate, generating inactive adduct derivatives classified as advanced peroxidation end products (ALEs) and advanced glycation end products (AGEs), respectively [200, 201] (Figure 2). In particular, lysine, histidine, and cysteine residues can react with lipid peroxidation products (HNE, MDA), through a Michael addition reaction, while lysine ε-amino groups can react with reducing sugars and their oxidative products, to generate several carbonyl derivates [202, 203].

AGEs are a heterogeneous group of molecules with carboxymethyl lysine, carboxymethyl valine, and pentosidine as the main protein products, while carboxymethyl lysine is a product of both lipid peroxidation and glycoxidation reactions [204–206].

AGEs increase with aging and their formation has been related to the level of carbohydrates; so, they have been linked to diabetes and obesity [207], as well as other diseases including atherosclerosis, Alzheimer's disease, and renal insufficiency [208, 209]. Mass spectrometry-based techniques represent a key method in identifying protein adducts and the specific site of modification but their use is still limited in routine clinical analysis [210, 211]. To address this, AGEs' assays are mostly based on the use of specific antibodies or spectrofluorimetric measurements based on the fluorescent properties of AGEs [212, 213]. Although promising results came from studies on skin autofluorescence in diabetic patients [214, 215], the serum fluorescence AGE (F-AGE) method did not distinguish women with gestational diabetes from the healthy controls [216].

The availability of polyclonal and monoclonal antibodies directed against different HNE-protein adducts (involving cysteine, lysine, or histidine residues) allowed the formulation of immunodetection methods which are commercially available. For example, specific antibodies are used to detect HNE-histidine adducts in tissues or biological samples and HNE-modified tau protein has been associated with neurofibrillary tangles in Alzheimer's disease [217].

The reliability of immuno-based methods is mostly dependent on the specificity of the antibodies utilized, that may lead to differences between the available commercial kits. A fructosamine assay for the detection of ketamine formed via a nonenzymatic glycation reaction of serum protein, and the HPLC measurement of furosine, a specific product obtained after hydrolysis of epsilon-amino-fructose-lysine, are also alternative biomarkers [218, 219].

Specific AGEs, as pentosidine and carboxymethyl lysine, can be measured by HPLC [220, 221]. However, their use as biomarkers and the development of specific assays in clinical application are hampered by the structural heterogeneity of these products, due to different mechanisms of formation, and because few AGEs have been characterized.

In addition to the role as marker of oxidative stress, the clinical relevance of AGE is indicated by their pathogenic role in immune- and inflammatory-mediated diseases.

First of all, the role of the receptor for advanced glycation end products (RAGE)-NF-kB axis in neuroinflammation is in line with the nonenzymatic glycosylation theory of aging, suggesting a central role of the AGEs in the age-related cognitive decline [17]. Besides, the soluble receptor for advanced glycation end products (sRAGE) plays an important role in the pathogenesis of the acute respiratory distress syndrome [222].

On the other hand, Turk et al. suggested a role for AGE-immune complexes in the pathogenesis of atherosclerosis. Compared to healthy subjects, both diabetic and nondiabetic patients with coronary artery disease had a higher concentration of circulating immune complexes containing the AGE moiety as antigen, whereas only diabetics had higher anti-AGE antibodies [223]. Autoantibodies to IgG-AGE were detected in patients with rheumatoid arthritis, suggesting that glycation of IgG results in the generation of new immunogenic epitopes, potentially inducing circulating autoantibodies [224]. Therefore, AGEs could be one of the links between metabolic syndrome and immune activation.

#### 3.3.2. Nitrotyrosine

3-nitro-tyrosine (3-NO-Tyr) is the main product of tyrosine oxidation which may occur either within a polypeptide or in free tyrosine residues. This modification can be generated through several pathways that include the reaction with ROS and RNS like ONOO^−^ and NO_2_^•^ [225–227] (Figure 2). NO^•^ generated by NOS can react with O_2_^•−^ to form ONOO^−^ that, at acidic pH, is present as protonated form (ONOOH) which is believed to decompose into HO^•^ and NO_2_^•^ to an extent of ~30% [10]. Generally, tyrosine oxidation is a two-step process with the formation of a tyrosine radical, generated by different oxidative steps, followed by the reaction with NO_2_^•^. Accurate determination of 3-NO-Tyr in biological samples requires gas or liquid chromatographic techniques coupled to mass spectrometry [228–230], conditions that are not feasible for high throughput in clinical analysis. For a better determination, protein extracts from biological samples can be completely hydrolyzed before quantification of 3-NO-Tyr by chromatography. A pitfall in this technique is the possible nitration of tyrosine residues in the sample by the presence of nitrite and the acid conditions during protein precipitation and hydrolysis [231].

ELISA assay based on specific antibodies are also available, but their use is limited by the different affinity of antibodies for different nitrated proteins and the low sensitivity [232, 233]. 3-NO-Tyr has been described as a stable marker of oxidative/nitrative stress in inflammatory diseases [234, 235], but its utility as clinical biomarker is still questioned. Some studies showed that 3-NO-Tyr plasma levels are increased in several conditions, such as asthma, diabetes, and cardiovascular diseases, and reduced following therapeutic treatments [236, 237]. Moreover, an involvement of 3-NO-Tyr in age-related neurodegenerative diseases has been suggested [238, 239].

#### 3.3.3. Advanced Oxidation Protein Products (AOPP)

The reaction of proteins with chlorinated oxidants such as hypochlorous acid results in chlorination of amino acid residues and formation of 3-chloro-tyrosine (3-Cl-Tyr) and 3,5-dichloro-tyrosine as main products. These oxidation products are generally classified as advanced oxidation protein products (AOPP) (Figure 2) and include protein aggregates by disulphide bridges and/or tyrosine cross-linking. AOPP is a marker of oxidative stress that reflects the chronic kidney failure and has been identified as a marker of inflammation in many diseases [240–250]. Chloro-tyrosine, as well as 3-nitro-tyrosine, can be produced by reaction with ipochlorous acid and ONOO^−^ both generated during inflammation, and it has been observed that AOPP may act as a mediator of the inflammation process and monocyte activation [240]. AOPP levels result as elevated in diseases such as diabetes, uremia, systemic sclerosis, atherosclerosis, and cardiovascular diseases and in patients with renal complications, increasing with the progression of chronic renal failure [241–244].

AOPP level can be measured by colorimetric tests using a chloramine standard or human serum albumin derivatives [245]. 3-Cl-Tyr is a highly specific biomarker that can be detected with very sensitive methods such as mass spectrometry [231, 246]. 3-Cl-Tyr has been detected in patients with atherosclerosis [247] and rheumatoid arthritis [248], in children with cystic fibrosis [249], and in the airways of preterm infants [250].

#### 3.3.4. oxLDL

Low-density lipoproteins can undergo oxidative modification, and this has been correlated with atherosclerosis and cardiovascular diseases [251, 252].

The most common test makes use of specific antibodies that recognize selected modifications of LDL amino acidic residues (i.e., aldehyde-modified lysine residues or oxidized phospholipid-modified residues). However, the use of oxLDL as a biomarker of oxidative stress has been criticized because of the heterogeneity of oxidation products, the low specificity of the antibodies, and the different results obtained depending on the assay utilized [253, 254].

In addition, the clearance of oxLDL and the formation of immunocomplexes must be taken into account. Patients with ischemic stroke with intracranial atherosclerosis had a higher baseline level of oxLDL and a greater decline after a standardized fat meal compared to those that presented extracranial atherosclerosis, indicating an increase of the clearance of the oxLDL after meal [255]. An increase in the uptake of oxLDL has been observed also in macrophages from type 2 diabetes (T2D) patients [256], potentially inducing foam cell formation and atherosclerosis. oxLDL may also induce maturation of dendritic cells and regulate the shift from classical (M1) to alternative (M2) macrophage activation and from T helper 1 (Th1) to T helper 2 (Th2) response, suggesting that these could act as a bridge between innate and adaptive immunity, involved in plaque development [27]. The Th2-induced response could account to the presence of anti-oxLDL antibodies in subjects with T2D and impaired glucose tolerance [257], as well as to the anti-MDA-LDL IgGs found in serum of patients undergoing off-pump and on-pump coronary artery bypass grafting [258]. Therefore, oxLDL are not only a marker of oxidative stress but also a pathogenic factor whose values should be evaluated in the context of a global clinical examination.

#### 3.3.5. Ischemia-Modified Albumin

Albumin, the most abundant protein in serum and other body fluids, is a carrier of many biomolecules. Albumin is susceptible to oxidation and carbonylation and may also act as an antioxidant system through the reversible oxidation of its cysteine residues. For this reason, it can be considered a general oxidative biomarker in several human diseases.

Myocardial ischemia results in structural changes to the N-terminus of the serum albumin related to the production of ROS [259, 260]. These changes reduce the ability of albumin to bind transition metals, particularly cobalt cations, which can be detected by the albumin cobalt-binding (ACB) test [261–263]. Besides the N-terminal cobalt-binding site, albumin contains two additional sites that are negatively modulated by fatty acids binding to albumin. Therefore, it has been hypothesized that the release of fatty acids in myocardial ischemia is responsible for the lower cobalt-binding capability [264]. The ABC test indirectly detects Ischemia Modified Albumin (IMA) by measuring the decreased binding capacity of albumin for cobalt [265] and has been carried out by the Food and Drug Administration (FDA) to detect myocardial ischemia. Growing evidence suggest that IMA is not only specific for cardiac ischemia, but its elevated levels are also reported in patients with liver cirrhosis, pulmonary embolism, diabetes mellitus, cerebrovascular disease, and Alzheimer's disease [266–269]. Thus, measurements of IMA serum levels could be a new marker of oxidative imbalance. However, ACB test is sensitive to pH changes, altering the metal-binding capacity of the albumin, as well as temperature and time of sample storage. Analysis should be performed within 2 h or the serum should be separated and frozen [270, 271]. Recently, several immunoassays based on specific antibodies anti-IMA have been introduced in the market.

## 4. Redox Proteomic and Markers Based on Cysteine and Redox Enzymes

The powerful strategy offered by the mass-proteomic approach makes it now possible to reach high sensitivity and specificity in determining oxidative modifications of selected proteins. If fact, redox proteomic can provide information on both the identification of the oxidized protein and the extent of oxidative damage occurring at the protein level [272–274]. Proteins may become reversible oxidized in response to a redox signalling, but irreversible oxidative modifications are associated with disorders and pathologies [275, 276]. Thus, a profile of oxidative modification of plasma or tissue sample proteins is a promising approach that will help in clinical determination of several human diseases and pathological states [189, 276, 277]. This will also make the identification of novel biomarkers and therapeutic targets for different human diseases possible.

In particular, components whose deregulation can result in oxidative stress, such as the ROS-generating enzymes, and antioxidant defence systems, which change in response to increased redox stress, can be used to assess the redox state of the body or specific tissues and cells in health and disease.

In the context of redox proteomic, major players are cysteine residues (including the GSH system), antioxidant (SOD, CAT, and GPX), and ROS-generating enzymes, as well as the transcription factors involved in their regulation [278] (Table 3).

Surface-exposed cysteine residues are particularly sensitive to oxidation by ROS and RNS and are the most vulnerable among all amino acids [240].

Although the reactivity of thiol groups toward H_2_O_2_ is very low, the nucleophilicity and reactivity toward several ROS species, including HO^•^, HClO, O_2_^•−^, and NO^•^, increase when the sulfur atom of the thiol group becomes deprotonated. Solvent exposure of the cysteine residue and the presence of neighbour polar residues exert a great influence on thiol group pKa. Thus, cysteine oxidation by ROS depends on the protein context and provides the basis for selective and specific modifications [279, 280].

**Figure 3:**
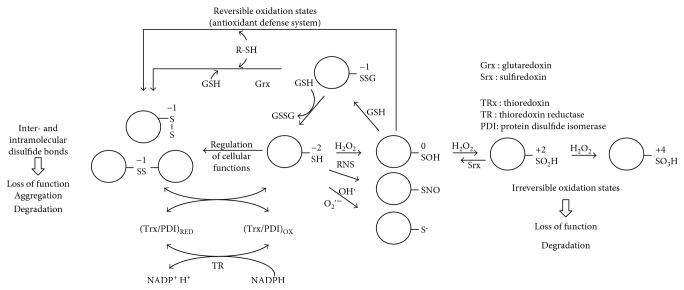
Reversible oxidation of protein cysteine residues. GSH: glutathione; H_2_O_2_: hydrogen peroxide; O_2_^•−^: superoxide; RNS: reactive nitrogen species; RS^•^: sulfur atom; −SO_2_H: sulfinic acid; −SO_3_H: sulfonic acid; −SOH: sulfenic acid.

The primary product of cysteine residue oxidation by H_2_O_2_ is the sulfenic acid (−SOH), whose stability and further reactivity may be influenced by the presence or availability of a proximal thiol group, resulting in the formation of a disulfide bond [281, 282] (Figure 3). Additionally, sulfenic acid may further react with H_2_O_2_ to produce sulfinic (−SO_2_H) and sulfonic (−SO_3_H) acids (Figure 3). Cysteine residues may also react with HO^•^ and O_2_^•−^ species, resulting in the formation of a highly reactive radicalic sulfur atom (RS^•^), which can further react with another thiol residue generating a disulfide, while the reaction with NO^•^ produces a S-nitrosylated cysteine [283].

Oxidation of cysteine residues is reversible, with the exception of sulfinic and sulfonic acids products; it may be reversed to the thiol form by reaction with GSH and/or specific enzymatic activities (thioredoxins, glutaredoxins, and protein disulfide isomerases) [284–286] (Figure 3).

The reversible protein oxidation is an important feature for the antioxidant defence systems, which can efficiently help in reducing the intracellular levels of oxidized proteins, produced upon cell exposure to damaging agents, and prevent the accumulation of misfolded or self-aggregating proteins [190, 273, 287–289] (Figure 3).

Reversible protein modifications may be also an important feature for signalling pathways involving ROS and RNS through the chemical modification of selected substrate proteins. This provides the basis for several redox-regulated cellular processes and enzymatic functions which imply redox-dependent modifications [290–292]. So, protein oxidative modifications can be a consequence of oxidative or nitrosative stress as well as the reflection of redox-regulated processes [273, 293].

### 4.1. Protein Glutathionilation

Reversible protein-S-glutathionylation can occur either under physiological conditions, within redox signalling pathways, or as result of GSH antioxidant activity through the reduction of oxidized cysteine residues and the formation of mixed disulfide protein-glutathione (PSSG). Cysteine- (SOH-) glutathionilation may act as a protective mechanism preventing further irreversible oxidation to sulfinic or sulfonic acids [294]. Reduction of PSSG can take place spontaneously, when the GSH/GSSG ratio is high, or can be catalyzed by protein thiol-disulfide oxidoreductases, such as glutaredoxins, protein disulfide isomerases, thioredoxin, peroxiredoxins, and sulfiredoxins [295]. Recent advances in redox proteomic techniques have led to the identification of many S-glutathionylated proteins and their involvement in redox-regulated pathways. Reversible protein-S-glutathionylation in monocytes and macrophages has emerged as a new and important signalling paradigm, which provides a molecular basis for the well-established relationship between metabolic disorders, oxidative stress, and cardiovascular diseases [296].

Measurement of S-glutathionylation of functional important proteins is a promising biomarker. However, this is hampered by complexity in the methodologies (accessing tissue samples and procedural artefacts) which requires special care in sample handling and preparation [297]. A simpler approach is analyzing S-glutathionylation of proteins in circulating cells. Glutathionylation of haemoglobin has been proposed as a marker of oxidative stress, and an increase in protein modification has been reported in patients with diabetes, hyperlipidaemia, and renal failure [298, 299].

Although S-glutathionylation can be easily measured by Western blotting under nonreducing conditions, the use of more effective approaches is required for an accurate quantification. MS techniques are valid but require specialized instrumentation. In addition, ELISA tests using monoclonal anti-glutathione antibody have been developed [300–302].

### 4.2. Glutathione and Cysteine

GSH is a tripeptide representing the most abundant nonprotein thiol present in the cell, where its concentration can reach the millimolar range [303, 304]. GSH acts as an antioxidant defense system by its ability to scavenge ROS through the reversible oxidation to GSSG (Figure 3). GSSG can be enzymatically reduced to GSH by the activity of glutathione reductase (GR) and the reducing power of NADPH. Glutathione is mainly stored within the cytosol, where the ratio GSH/GSSG is ranging from 30 to 100 [305]. This ratio is ten times lower in the serum and in the endoplasmic reticulum and decreases in the presence of oxidative stress. Glutathione synthesis depends on the availability of cysteines, the rate-limiting precursor, and this makes its use as a marker of oxidative stress questionable. Besides, diurnal variation in GSH and cysteine has been reported [306]. However, several studies relate the GSH levels and GSH/GSSG ratio to pathological conditions [254]. The measurement of GSH, GSSG, and their ratio in blood has been considered an index of the redox status in the whole-organism and a useful marker of diseases in humans [307, 308]. Several methods have been used to determine the GSH in biological samples (spectrophotometry, HPLC, capillary electrophoresis, nuclear magnetic resonance, and mass spectrometry) [307]. However, GSH and its oxidate form GSSG do not represent powerful biomarkers of oxidative stress because of some methodological artifacts. For instance, sample acidification for protein precipitation leads to an increase in GSSG levels [308].

### 4.3. Nrf-2 and NF-kB

**Figure 4:**
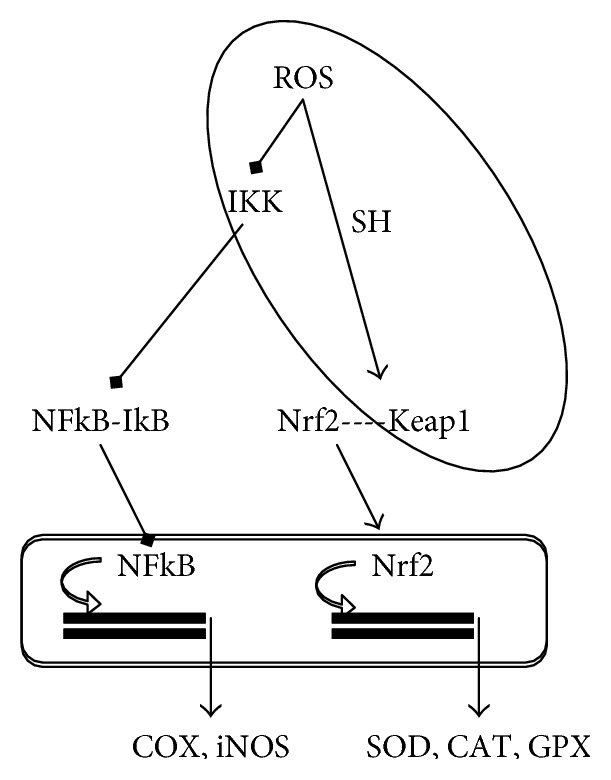
Cysteine-regulated gene expression. CAT catalase; COX: cyclooxygenase; GPX: glutathione peroxidase; IKK: I*κ* kinases; iNOS: inducible nitric oxide synthase; Keap1: Kelch-like ECH-associating protein 1; Nfr2: nuclear factor-erythroid 2-related factor 2; NF-kB: nuclear factor kappa-light-chain-enhancer of activated B cells; ROS: reactive oxygen species; SH: thiol; SOD: superoxide dismutase.

As mentioned above, oxidation of selected cysteine residues present in specific proteins may result in the regulation of cellular response to oxidative stress. This is the case for Nrf-2, a conserved transcription factor that is a master regulator of the antioxidant response system controlling the expression of more than 250 genes. Nrf-2 is normally sequestered into the cytoplasm complexed to the protein Keap1 (Kelch-like ECH-associating protein 1), which facilitates its polyubiquitination and proteasome-mediated degradation [309]. Keap1 contains specific cysteine residues sensitive to oxidation in the presence of oxidants or other electrophiles (Figure 4). Thus, Keap1 functions as a specific sensor of stress that upon oxidation, and resulting conformational change, releases Nrf-2 allowing its translocation into the nucleus.

Nrf-2 promotes the transcriptional activation of a specific set of target genes containing the antioxidant response elements (AREs) in their promoter regions and encoding antioxidant and detoxifying enzymes (i.e., glutathione S-transferase, glutathione synthetase, heme oxygenase 1, and NAPH-oxidoreductase) (Figure 4). Thus, Nrf-2 is related to the cellular defence against ROS and it has been observed that its activity declines with age as well as with degenerative disorders [310].

On the other hand, an increased Nrf-2 activity has been observed in transformed cells [311], where it provides a reduced sensitivity both to the large amounts of ROS generated during the active proliferation and to chemotherapeutic drugs, whose enzymatic elimination requires enhanced levels of NAPDH. For these reasons, Nrf-2 can be considered a valid biomarker and its levels in tumour samples, quantified by immunological methods or by RT-PCR, may have a clinical significance. Recently, the determination of Nrf-2 levels, in combination with measuring high-mobility group box-1 (HMGB1) expression, might represent a useful tool in the early detection of post-trauma complications [312].

Whereas Nrf2 has a primary role in antioxidant enzymes gene expression, NF-kB is involved in the transcription of ROS-generating and inflammatory enzymes (Figure 4). As observed for Nrf2, some cysteine residues are involved in the translocation of NF-kB to the nucleus (Figure 4). In particular, cysteine 179 of I*κ* kinases (IKK) is a target of the S-glutathionylation-induced inactivation and glutaredoxin reverses this effect [313]. Furthermore, electrophilic modifications of cysteine 179 of IKK inhibit NF-kB activation and have been suggested as one of the mechanisms involved in the anti-inflammatory and COX-inhibitory effects of nutraceuticals [314, 315]. Similarly, antioxidants with catechol and electrophilic moieties induce the Nrf2-mediated gene expression of antioxidant enzymes acting as pro-oxidants rather than antioxidants [316, 317].

### 4.4. Enzymes

**Figure 5:**
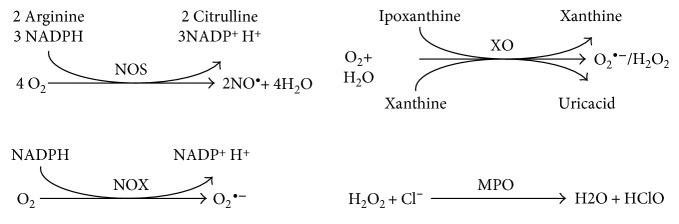
ROS generating enzymes. H_2_O_2_: hydrogen peroxide; HClO: hypochlorous acid; MPO: myeloperoxidase; NOS: NO synthase; NOX: NADPH oxidase; O_2_^•−^: superoxide; XO: xanthine oxidase.

ROS-generating enzymes are involved in several cell functions and their alteration may result in imbalanced redox status (Figure 5).

The established role in diseases of XO [318] and NOX [319, 320] suggested their pharmacological inhibition in the prevention and treatment of pathologies related to oxidative stress.

Some ROS-generating enzymes can be found in the circulation and thus can be used as markers of oxidative stress, such as NOS and NOX (Figure 5) involved in oxidative burst.

It has been shown that high levels in the circulations of MPO, a heme peroxidase abundant in granules of human inflammatory cells, which catalyzes the conversion of H_2_O_2_ to HClO with the production of ROS (Figure 5), are associated with cardiovascular disease [321], chronic obstructive pulmonary disease [322], and Alzheimer's disease [323].

Oxidant species derived from MPO lead to the production of specific oxidation products, such as 3-Cl-Tyr. This can be used as biomarker in several diseases [324], as above described, and its levels correlate with MPO. However, expensive equipment are required to detect the levels of MPO-dependent specific biomarkers and this represents a limitation in their use. Moreover, the concentration of these biomarkers in biological samples is low, which complicates accurate measurement.

XO catalyzes the oxidation of hypoxanthine and xanthine to UA in the terminal steps of purine nucleotide metabolism [325], which also leads to the production of O_2_^•−^ [326] (Figure 5). Given that XO produces ROS in stoichiometric quantities along with UA, it represents one of the major sources of free oxygen radicals in human physiology. Upregulation of XO activity may lead to an increase in UA serum levels, oxidative stress, and endothelial dysfunction [327–329]. XO exists in two interconvertible forms, XO (that oxidizes xanthine to UA using oxygen as the electron acceptor and produces superoxide or H_2_O_2_) and xanthine dehydrogenase (XDH) (that carries out the same reaction but uses NAD^+^ and generates NADH). XDH is the predominant form in well-oxygenized tissue [330], but it can be converted to XO under various conditions [331, 332]. Inflammatory or hypoxic conditions promote XDH expression in tissues and vascular endothelial cells and stimulate XDH release into the circulation [333]. Once in the circulation, XDH is quickly converted, by reversible oxidation of the sulfhydryl residue or by irreversible proteolysis, into XO which binds to the endothelial surface, resulting in amplified XO-derived ROS formation [334]. This XO-induced oxidative stress has been detected in renal and cardiovascular diseases, such as heart failure, chronic obstructive pulmonary disease, pulmonary hypertension, sickle cell disease, and diabetes [334]. An increase in XO activity has been reported in patients with heart failure [326, 335], whereas XO activity and its plasma levels are raised in presence of inflammatory agents and interferon [336] and seems to play a key role in ischemia-reperfusion injury [337].

As described for MPO, an indicator of the enzyme activity in vivo could be the detection of a metabolite or a reaction product. Serum levels of UA may reflect XO activity, but they are also dependent on dietary intake, and purine metabolism, and renal filtration and reabsorption, as well as endothelia dysfunctions. Higher UA levels are associated with metabolic, cardiovascular, and renal abnormalities, and UA has been recently proposed as a biomarker and therapeutic target in diabetes [338–340]. UA is a powerful antioxidant in plasma and can scavenge O_2_^•−^ and HO^•^, and allantoin is its oxidative product of which formation is independent of changes in UA levels [341–343]. This makes allantoin a promising biomarker of oxidative status, considering also its stability regardless of the storage or sample preparation, but its quantitative determination requires specific instrumental techniques as liquid/gas chromatography and mass spectrometry [344–346].

**Figure 6:**
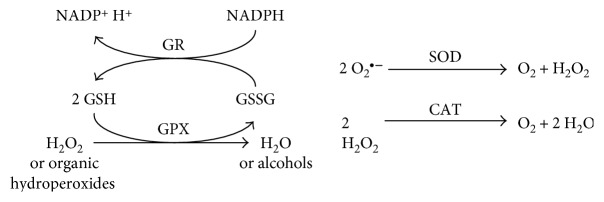
Antioxidant enzymes. CAT: catalase; GPX: glutathione peroxidase; GR: glutathione reductase; H_2_O_2_: hydrogen peroxide; O_2_^•−^: superoxide; SOD: superoxide dismutase.

The most important antioxidant enzymes are SOD, CAT, and glutathione-dependent enzymes, such as GPX, GR, and glutathione transferases (GSTs) (Figure 6).

SODs are a family of enzymes catalyzing dismutation of superoxide into oxygen and H_2_O_2_. There are three isoforms of SOD, with a different cellular localization and metal cofactor: homodimeric Cu/Zn-SOD localized in the cytosol and in the mitochondrial intermembrane space, homotetrameric Cu/Zn-SOD with an extracellular distribution, and homotetrameric Mn-SOD localized in the mitochondria [347]. SOD acts also as pro-oxidant producing H_2_O_2_; therefore, other antioxidant enzymes such as CAT and GPX are required and an imbalance in their ratio may be dangerous.

SOD activity can be measured analyzing the inhibition in the rate of reduction of a tetrazolium salt by O_2_^•−^ generated through a xanthine/XO enzymatic system [348, 349].

CAT, which catalyzes the conversion of H_2_O_2_ into water and oxygen, is a homotetrameric protein containing four iron heme and largely located in the peroxisomes. CAT activity can be measured by several colorimetric/spectrophotometric assays [349, 350].

GSH redox cycle is regulated by GPX and GX. GPXs are a family of selenium-dependent isozymes that catalyze the reduction of H_2_O_2_ or organic hydroperoxides to water and alcohols through the oxidation of GSH to GSSG. GR then reconverts GSSG to GSH using the reducing power of NADPH [351]. GPX activity can be measured using cumene hydroperoxide and GSH as substrates in a coupled reaction with GR [352]. The GSSG formed after reduction of hydroperoxide is recycled to its reduced state by GR in the presence of NADPH. The oxidation of NADPH is accompanied by a decrease in absorbance at 340 nm proportional to GPX activity. GR activity can be measured in a similar manner using GSSG and NADPH as substrates [353].

Differently from ROS-generating enzymes, conflicting results came from human studies that evaluated the relationship between diseases or ageing and antioxidant enzymes. Despite meta-analyses suggesting that polymorphisms of antioxidant enzymes are associated with T2D [354] and hypertension [355], decreased or increased activities (or levels) have been reported for SOD, catalase, GPX, and/or GR in these diseases [356–367]. Activity of SOD or CAT was significantly higher in elderly hypertensives [356] and T2D [360, 361, 368] when compared with healthy controls. Increased SOD activity has been reported also in coronary artery disease patients [369] and in women with the polycystic ovary syndrome [370]. In patients with Crohn's disease, SOD and GPX increase during the active phase and return to normal during the remission phase [371]. It has been suggested that the increase in antioxidant enzymes may represent a compensatory upregulation in response to increased oxidative stress [361, 368]. Results of Karaouzene et al. suggest that this response depends on age [372]. Erythrocyte SOD and CAT activities were enhanced in obese young patients but reduced in obese older men [372]. The ARE/Nrf2 pathway is the major player in the induction of the expression of antioxidant genes [309]. However, although phytochemicals contained in fruits and vegetables are known to induce Keap1/Nrf2 system [373] in a meta-analysis [374] of randomized controlled trials, no significant differences were observed between fruit or vegetable juices and placebo in SOD and CAT, despite the reduction of MDA.

In order to understand the contrasting results in human studies, some methodological considerations must be made. Conventional methods for measuring enzymes are enzyme activity, protein content (Western blots and immunological techniques), or gene expression (reverse-transcription polymerase chain reaction (RT-PCR)) (Table 3) [129]. First of all, it must be taken into account that different samples have different antioxidant content. In a meta-analysis, decreased activities of SOD and GPX were observed in plasma/serum of postmenopausal women with osteoporosis, but the activities of SOD in erythrocytes and of CAT in plasma/serum were not statistically different from the control group [375]. Concerning the measure of cellular enzymes, it must be considered that processing and cryopreservation procedures could affect peripheral blood mononuclear cell (PBMC) gene expression [376, 377]. In addition, PBMC exclude from the analysis the neutrophils that are the major component of the full blood count [378], reducing the clinical relevance of this sample compared to whole blood RNA. On the other hand, the different cell types present in blood have a different content of enzymes. In whole-blood iNOS, RNA was expressed predominantly in monocytes [379]. Although the presence of MPO in lymphocytes has been recently reported, it is very low compared to neutrophils/monocytes [380]. Concerning antioxidant enzymes, neutrophils have higher levels of SOD and catalase transcripts compared to monocytes [381]. On the other hand, GSH content and GPX transcript and activity are higher in monocytes [381]. In this context, results from meta-analysis document that neutrophil-to-lymphocyte ratio [382–387] and lymphocyte-to-monocyte ratio [388, 389] were related to clinical oncological outcomes in cancer patients. Also, coronary artery disease is associated with altered ratio of leukocytes [390], the expansion of monocytes, and the reduction of the CD4/CD8 T cell ratio, and B cell lymphopenia can be observed in end-stage renal disease [391]. Furthermore, also in heathy subjects, the normal ranges of the different leukocyte populations are very large [392]. Probably, the use of cell marker coding genes (CD4, CD8, CD14, etc.) as housekeeping genes could normalize the results for the physiologically or pathologically different content of cells between subjects [378]. This approach could also help in conditions, such as hyperglycemia, that can influence the expression of housekeeping genes [393].

## 5. Measuring the Nonenzymatic Antioxidant Capacity in Body Fluids

The nonenzymatic antioxidant capacity (NEAC), also named total antioxidant capacity (TAC), is defined as the moles of oxidants neutralized by one liter of body fluids [278, 394–396]. In plasma, nonenzymatic antioxidants include endogenous (e.g., UA, bilirubin, and thiols) and nutritional (e.g., tocopherols, ascorbic acid, carotenoids, and phenolics) compounds [278, 394]. Various assays for NEAC [129, 397–412] measure either their radical scavenging or reducing capacity. Reaction mechanisms include hydrogen atom transfer (HAT) and single electron transfer (SET) (Table 4). The latter reports on antioxidants' reductive capacity, including its metal reducing power, and could be considered an “indirect assay,” whereas the former is a “direct assay” (competitive) in which the inhibition of the oxidation of an indicator substance is determined as a measure of the antioxidant capacity [406, 407]. The most common HAT methods are oxygen radical antioxidant capacity (ORAC) and the total radical-trapping antioxidant parameter (TRAP), performed in aqueous solutions with 2,2′-azobis(2-methylpropionamidine) dihydrochloride (AAPH) as a thermolabile stoichiometric and water-soluble azo-radical generator (Table 4).

The Crocin bleaching assay can be performed under both hydrophilic and lipophilic conditions by using AAPH or 2,2′-azobis 2,4-dimethylnaleronitrile (AMVN), which is AAPH's lipophilic equivalent. Aldini et al. [401] monitored the oxidation of the lipid compartment of plasma by using 2,2′-azobis(4-methoxy-2,4-dimethylvaleronitrile) (MeO-AMVN), as lipid soluble radical initiator and C11-BODIPY^581/591^ as lipophilic fluorescence probe. The MeO-AMVN-C11-BODIPY^581/591^-based total antioxidant performance (TAP) assay was reported to be sensitive to plasma antioxidants localized in both the lipophilic and hydrophilic compartments [400]. In all HAT methods, ROO^•^ reacts with the target compound resulting in changes of fluorescence or absorbance of probe (Table 4).

Area under the curve (AUC), lag phase, or Stern-Volmer-like relation are used in order to measure the competition reaction and the standard antioxidant Trolox is used as reference (Table 4). NEAC values are reported as Trolox equivalents (TEAC) also in the total antioxidant status (TAS) assay and in the competitive (Randox) SET-based 2,2′-azino-bis(3-ethylbenzothiazoline-6-sulphonic acid (ABTS) assay, both based on the production of HO^•^ via Fenton reaction [399]. In other SET assays, using the stable radical cation ABTS^•+^ or 2,2-diphenyl-1-picrylhydrazyl (DPPH), the target compound extracts an electron from the antioxidant and changes color (Table 4). In these assays, it is assumed that antioxidant activity is equal to reducing capacity [406]. Other SET methods measure the reducing power of antioxidants through redox reaction with iron (ferric reducing antioxidant potential (FRAP)) or copper (cupric reducing antioxidant capacity (CUPRAC)) (Table 4). The latter has been applied to both lipophilic and hydrophilic fractions of serum [398]. However, as for Crocin bleaching assay, bilirubin and carotenoids that absorb at the wavelength of determination could interfere with the results (Table 4). Similarly, the oxidation product of bilirubin (biliverdin) absorbs at the wavelength of determination of FRAP method (Table 4). Although NEAC assays present the advantage of integration of the individual antioxidant actions of different compounds and their additive, synergistic, or antagonistic interactions, many limitations have been pointed out previously [129, 403–407] (Table 4). Different NEAC assays can give different results, both in disease states [396] and after dietary supplementation with antioxidant-rich plant foods and beverages [394, 412, 413]. In a meta-analysis, Lettieri et al. [394] reported that TRAP, ORAC, and FRAP, but not TEAC, displayed an increase in plasma NEAC in both acute and chronic studies. From that, the authors [394] suggested that FRAP could be more sensitive than the TEAC assay within the SET methods to assess plasma NEAC. Accordingly, Carrión-García et al. [414] found a statistically significant positive correlation between plasma FRAP and dietary FRAP, either derived from the food frequency questionnaire (FFQ) and/or from a 24-hour recall (24-HR), whereas plasma ORAC without proteins, but not plasma ORAC, was related with 24-HR-based dietary ORAC, suggesting that proteins rather than dietary antioxidants have a primary role in plasma antioxidant defences. Despite FRAP appearing to be the more sensitive method to evaluate the effects of antioxidant-rich foods on NEAC, it must be taken into account that reduced iron is the major player in the Fenton reaction. Therefore, an increase in the iron reducing power could be more likely detrimental than beneficial in conditions of high levels of iron and low levels of antioxidant enzymes [364, 396, 415–420]. On the other hand, an increase in antioxidant enzymes as adaptive response to oxidative stress has been observed in T2D [358, 359, 363, 368]. Simultaneously with increased MDA levels, significantly higher activities or levels of SOD and/or CAT were found [358, 359, 363, 368]. Some studies reported unchanged or decreased CAT and/or GPX and elevated SOD and lipoperoxidation markers in T2D [360, 361, 412] and CVD [369, 421]. The balance between SOD and CAT and/or GPX dictates H_2_O_2_ levels that may potentially react with reduced metals. As previously pointed out, in NEAC assays the contribution of the antioxidant enzymes is neglected [405, 406]. Therefore, the lower total antioxidant status in these cases must be interpreted with caution [421, 422].

Despite a correspondence between the effect on F2-IsoPs (the golden standard of oxidative stress) and NEAC has been reported in 67% (14/21) of the interventions with foods and in 77% (10/13) of the interventions with galenics [413] of human studies in a systematic review, in the majority of the cases the correspondence was the lack of change for both biomarkers, whereas increases in NEAC and decreases in F2-IsoPs were observed only in 9.5% (2/21) of interventions with foods and 30.7% (4/13) of interventions with supplements [413]. Furthermore, despite gas chromatography mass spectrometry or liquid chromatography mass spectrometry techniques giving a more reliable and precise measure of F2-IsoPs, in the majority of these studies (5/6), enzyme-linked immunosorbent assay-based methods were used [413]. Last, but not least, in one of these studies [423], the increase in NEAC and the decrease in F2-IsoPs were not associated with lipid and glucose metabolism markers, nor with renal and liver functionality markers in uremic patients after 4 weeks of supplementation with Emblica officinalis extract, suggesting a low clinical relevance of NEAC in certain conditions. In this context, the major bias of all methods is that, despite hyperuricemia being detrimental and associated with CVD [424–426], UA is the major contributor of NEAC measured in plasma (60–80%), saliva (70%), and urine (75%) [395]. In case-control studies, there was an accordance between UA concentration and NEAC, as well as between salivary or urinary NEAC and plasma or serum NEAC [395]. On the other hand, only in 44% of the interventions with antioxidant foods, beverages or supplements urinary NEAC was related to UA, probably due to the excretion of phenolic metabolites.

In order to avoid the UA interference, methods for UA independent NEAC have been proposed in both plasma and urine [427–430]. In particular, the consumption of 500 g of strawberries daily for 9 days had no effect on circulating phenolics and plasma NEAC, whereas it increased UA-independent NEAC and urinary metabolites of polyphenols [431]. Furthermore, it has been suggested that urinary UA-independent NEAC normalized for creatinine could provide more reliable information about the antioxidant status in children and adults with Down syndrome [429].

Although UA-independent NEAC could be a good approach also for salivary NEAC, it has been observed that salivary NEAC was affected by emotional and psychological factors [432]. The latter could induce hyperactive sympathetic nervous system and the activation of platelets [433], potentially changing the plasma NEAC. In this context, it has been suggested that plasma and not serum should be preferred for NEAC measurement, in order to avoid ROS generation by platelets during processing (aggregation) [434]. However, platelets' activation can occur during the time course of NEAC methods in plasma samples and, alternatively, vigorous vortexing produces platelets microparticles further confounding the results.

In fact, sample type, collection, processing, and methodological limitation must be taken into account when measuring NEAC. Despite the fact that the use of a refrigerated microcentrifuge to rapidly prepare plasma could avoid any thermal stress and instability of antioxidants in biological samples [434], recent results indicate that centrifugation at room temperature is the preferred option for many applications, giving lower microparticles and less hemolysis in plasma [435]. Hemolysis could bias many NEAC methods, and the presence of platelets and microparticles in the reaction mixture could affect the results. Considering that microparticle count is lower in serum compared with plasma after centrifugation at both room temperature (1.73 × 10^7/ml versus 3.72 × 10^7/ml) and 4°C (1.33 × 10^7/ml versus 7.4 × 10^7/ml) [435], probably serum and not plasma could be the better sample for NEAC evaluation.

On the other hand, although NEAC of saliva or urine has led to increasing interest, due to simple and noninvasive collections, many factors could give spurious results. In particular, blood contamination, periodontal diseases, bacterial counts and flow rate must be evaluated in order to avoid misinterpretation of the results of salivary NEAC and normalize for dilution [395, 436]. Also, urinary samples require normalization for dilution [395]. Despite results are normalized for the creatinine excretion, age, sex, muscle mass, renal diseases, and diet all have an influence on creatinine excretion [395].

From the mentioned above limitations and potential confounding, it appears that a clear association between increase in NEAC and health benefit is difficult to evaluate [405, 406]. Therefore, as previously suggested, each study requires a careful design of the experimental protocol and caution should be taken in the interpretation of results.

## 6. Conclusion

A clinically useful biomarker, besides being correctly measured, must be diagnostic, have prognostic value, and correlate with the disease degree. It must also be reasonably stable, present in an easily accessible specimen, and its measurement should be cost-effective.

In order to evaluate the redox status in particular conditions (smoking habit, disease states), ex vivo free-radical production and oxidative stress in body fluids are measured. These methods are used also in human intervention studies to associate the levels of ingested antioxidants (by foods or supplements) with improvement of the body antioxidant status. Despite the fact that it has been suggested that nutraceuticals are capable of improving health, significant methodological bias must be taken into account in the interpretation of data from the measurement of reactive species in leukocytes and platelets by flow cytometry, from the evaluation of markers based on ROS-induced modifications, from the assay of the enzymatic players of redox status, and from the measurement of the total antioxidant capacity of human body fluids.

It has been suggested that the bias of each method could be overcome by the evaluation of oxidative stress by using more than one criterion [129, 404]. In this context, indexes of redox status have been proposed [437, 438].

The OXY-SCORE [437] was computed by subtracting the protection score (GSH, alpha- and gamma-tocopherol levels, and antioxidant capacity) from the damage score (plasma free and total malondialdehyde, GSSG/GSH ratio, and urine F2-IsoPs). The oxidative-INDEX [438] was calculated by subtracting the OXY (the antioxidant capacity measured with the OXY adsorbent test) standardized variable from the ROM (the reactive oxygen metabolites measured with the d-ROM) standardized variable.

These scores are related to CVD, age, gender, and smoking habit [437–442]. The oxidative-INDEX has been successfully used also in case-control studies (liver diseases and cancers) [443–445] and in a human intervention study with antioxidant [446].

More recently, a multiple factor analysis (MFA) that allows for simultaneous analysis of multiple parameters, classified according to their physiological meaning in athletes following strenuous endurance exercise, was applied [447]. This integrative approach reveals a close relationship between the oxidative index, the inflammatory IL-8, and the cardiac marker N-terminal pro-B-type natriuretic peptide (NT-proBNP). Athletes that showed a higher improvement of the oxidative index after the race, presented small changes in NT-proBNP and IL-8 levels, whereas subjects with minimal variation in the oxidative index had a marked postrace increase in NT-proBNP and IL-8 concentrations.

On the other hand, in some diseases, the choice of the markers that must be considered in the global index should dictate the clinical relevance in the subjects selected. Condezo-Hoyos et al. [448] measured an array of oxidative stress biomarkers (SH, GSH, UA, ORAC, MDA-bound protein, protein carbonyls, AOPP, 3-nitrotyrosine, CAT, XO, and MPO) in patients with chronic venous insufficiency (CVI) and used for the OXyVen index calculation the normalized and standardized plasma parameters which showed a significant statistical difference between CVI patients and controls (SH, MDA-bound protein, protein carbonyls, and CAT activity).

In conclusion, the clinical significance of biomarkers of oxidative stress in humans must come from a critical analysis of the markers that should be dictated by the study aim and design and should give overall an index of redox status in particular conditions.

## Figures and Tables

**Table 1:** Fluorescent probes used for the measurements of reactive oxygen and nitrogen species by flow cytometry.

Probe (localization)	ROS/RNS	Fluorescence	Leukocytes	Platelets	Limitations and confoundings
DCFH-DA (intracellular)	HO^•^ ONOO^−^ ROO^•^ NO_2_^•^ Indirect H_2_O_2_	↑ green (DCF)	Yes	Yes	Hemolysis Self-propagation of DCF radicals MDR substrates or inducers Esterase inhibitors Plasma esterase in whole blood or PRP EDTA and citrate Antioxidants
DAF-2 DA/DAF-FM DA (intracellular)	NO^•^	↑ green (DAF-Ts)	Yes	No	MDR substrates or inducers Esterase inhibitors Plasma esterase in whole blood
DHR123 (intracellular)	HClO H_2_O_2_ ONOO^−^	↑ green (Rho123)	Yes	No	Self-propagation of DHR radicals MDR substrates or inducers Antioxidants
HE (intracellular)	O_2_^•−^	↑ red (ethidium)	Yes	No	Intercalating agents
C11-BODIPY^581/591^ (membrane)	HO^•^ ROO^•^	Shift from red to green	Yes	Yes	Hemolysis Antioxidants

C11-BODIPY^581/591^: 4,4-difluoro-5-(4-phenyl-1,3-butadienyl)-4-bora-3a,4a-diaza-s-indacene-3-undecanoic acid; DAF-2 DA: 4,5-diaminofluorescein diacetate; DAF-FM DA: 4-amino-5-methylamino-2′,7′-difluorofluorescein diacetate; DAF-Ts: triazolofluoresceins; DCF: 2′,7′-dichlorofluorescein; DCFH-DA: dihydrochlorofluorescein diacetate; DHR123: dihydrorhodamine 123; EDTA: ethylenediaminetetraacetic acid, H_2_O_2_: hydrogen peroxide; HClO: hypochlorous acid; HE: hydroethidine; MDR: multidrug resistance; NO^•^: nitrogen monoxide; NO_2_^•^: nitrogen dioxide; O_2_^•−^: superoxide radical; HO^•^: hydroxyl radical; ONOO^−^: peroxynitrite; PRP: platelet-rich plasma; Rho123: rhodamine 123; ROO^•^: peroxyl radicals.

**Table 2:** Markers based on ROS-induced modifications.

Markers	Methods	Limitations and confoundings
*Lipid oxidation*		
HNE	HPLC, GC-MS Immunoassay	
MDA, alkenals, alkadienals	Spectrophotometric/fluorimetric (TBARS), HPLC (UV or fluorescence)Immunoassay	Sugars, amino acids, bilirubin and albumin, hemolysis
F2-IsoPs	Gas/liquid chromatography coupled with mass spectroscopy techniques Immunoassay	Hemolysis Antibody specificity
*DNA oxidation*		
8oxodG, 5-chlorocytosine, 5-chlorouracil, *ε*dA, *ε*dC	ELISA assays, HPLC-ECD, HPLC/GC-MS	Antibody specificity
*Protein oxidation*		
ALEs, AGEs	HPLC, Western blot after one-dimensional or two-dimensional electrophoretic separation, immunohistochemistry, ELISA	Structural heterogeneity of these products Antibody specificity
Carbonils	Spectrophotometric, HPLC, ELISA	
3-NO-Tyr	HPLC/GC-MS, ELISA	Possible nitration of tyrosine residues in the sample by the presence of nitrite and the acid conditions during protein precipitation and hydrolysis Antibody specificity
AOPP	MS, colorimetric assays	
oxLDL	Immunodetection (ELISA)	Antibody specificity
IMA	ABC test, immunodetection (ELISA)	Sensitive to pH changes, temperature, and time of sample storage Antibody specificity

8oxodG: 7,8-dihydroxy-8-oxo-2′-deoxyguanosine; ABC test: binding capacity of albumin for cobalt; AGEs: advanced glycation end products; ALEs: advanced lipoxigenation end products; AOPP: advanced oxidation protein products; F2-IsoPs: F2-isoprostanes; GC: gas chromatography; HNE: 4-hydroxy-2-nonenal; HPLC: high-performance liquid chromatography; ECD: electrochemical detection; IMA: ischemia-modified albumin; MS: mass spectroscopy; MDA: malondialdehyde; TBARS: thiobarbituric acid reactive substances.

**Table 3:** 

*Reversible cysteine modifications*	*Methods*	*Limitations and confoundings*
S-glutathionylation GSH/GSSG SH	MS, ELISA, WB Spectrofotometric	For an accurate quantification, a specialized instrumentation is required
*ROS-regulated transcription factors*	*Methods*	*Limitations and confoundings*
Nrf-2, NF-kB	Immunological techniques, RT-PCR	
*ROS-generating enzymes*	*Methods*	*Limitations and confoundings*
NOX, MPO, XO, NOS	Immunological techniques, WB, PCR, RT-PCR, enzymatic	Antibody specificity Different percentages of leukocytes' populations
*Antioxidant enzymes*	*Methods*	*Limitations and confoundings*
SOD, CAT, GPX, GR	Immunological techniques WB, PCR, RT-PCR, enzymatic	Antibody specificity Different percentages of leukocytes' populations

CAT: catalase; GPX: glutathione peroxidase; GR: glutathione reductase; GSH: glutathione; MPO: Myeloperoxidase; MS: mass spectroscopy; NOS: nitric oxide synthases; NOX: NADPH oxidase; PCR: reverse-transcription polymerase chain reaction; SOD: superoxide dismutase; WB: Western blot; XO: xanthine oxidase.

**Table 4:** Common used methods for NEAC measurements.

Method	Reaction and quantification	Limitations and confoundings
HAT ORAC	AAPH—induced: R-phycoerytherin (red) or fluorescein (green) fluorescence decay Competitive reaction kinetic, AUC	Lipophilic antioxidants not included Proteins
HAT TRAP	AAPH—induced: R-phycoerytherin fluorescence decay (red) DCFH ➔ DCF fluorescence increase (green) Competitive reaction kinetic, lag phase	Lipophilic antioxidants not included Not all the antioxidants give a lag phase Self-propagation of DCF radicals
HAT Crocin bleaching	AAPH- or AMVN-induced absorbance decay (450 nm) Competitive reaction kinetic, Stern-Volmer-like relation	Bilirubin and carotenoids that absorb at the wavelength of determination
HAT TAP	MeO-AMVN induced C11-BODIPY fluorescence increase (green) Competitive reaction kinetic, AUC	
TAS	Fenton reaction-induced dianisidyl radical absorbance increase (444 nm) Competitive, endpoint, TEAC	
SET (Randox)	Fenton reaction-induced ABTS radical formation (734 nm) Competitive reaction, endpoint, TEAC	
SET ABTS^•+^	Absorbance decay (734 nm) Noncompetitive, endpoint, TEAC	
SET DPPH^•^	Absorbance decay (515 nm) Noncompetitive, endpoint, EC50	Carotenoids that absorb at the wavelength of determination
SET FRAP	Absorbance increase (593 nm) Noncompetitive, endpoint	SH not included Biliverdin absorb at the wavelength of determination
SET CUPRAC	Neocuproine absorbance increase (450 nm). Noncompetitive, endpoint	Bilirubin and carotenoids that absorb at the wavelength of determination

AAPH: 2,2′-azobis(2-methylpropionamidine) dihydrochloride; ABTS: 2,2′-azino-bis(3-ethylbenzothiazoline-6-sulphonic acid); AMVN: 2,2′-azobis 2,4-dimethylnaleronitrile; AUC: area under the curve; CUPRAC: copper-reducing assay; DCFH: 2′,7′-dichlorodihydrofluorescein; DPPH: 2,2-diphenyl-1-picrylhydrazyl; EC50: efficient concentration (EC), the amount of antioxidant necessary to decrease by 50%; FRAP: ferric reducing antioxidant power; HA: T hydrogen atom transfer; MeO-AMVN: 2,2′-azobis(4-methoxy-2,4-dimethylvaleronitrile); NEAC: nonenzymatic antioxidant capacity; ORAC: oxygen radical antioxidant capacity; SET: single electron transfer; SH: thiols; TAC: total antioxidant capacity; TAP: total antioxidant performance; TAS: total antioxidant status; TEAC: Trolox equivalent antioxidant capacity; TRAP: total radical-trapping antioxidant parameter.
